# Diabetes in a TB and HIV-endemic South African population: Analysis of a virtual cohort using routine health data

**DOI:** 10.1371/journal.pone.0251303

**Published:** 2021-05-07

**Authors:** Tsaone Tamuhla, Joel A. Dave, Peter Raubenheimer, Nicki Tiffin

**Affiliations:** 1 Division of Computational Biology, Integrative Biomedical Sciences Department, Faculty of Health Sciences, University of Cape Town, Cape Town, South Africa; 2 Division of Endocrinology, Department of Medicine, Faculty of Health Sciences, University of Cape Town (UCT), Cape Town, South Africa; 3 Wellcome Centre for Infectious Disease Research in Africa, Institute of Infectious Diseases and Molecular Medicine, University of Cape Town, Cape Town, South Africa; 4 Centre for Infectious Disease Epidemiology Research, School of Public Health and Family Medicine, University of Cape Town, Cape Town, South Africa; 1. IRCCS Neuromed 2. Doctors with Africa CUAMM, ITALY

## Abstract

**Background:**

It is widely accepted that people living with diabetes (PLWD) are at increased risk of infectious disease, yet there is a paucity of epidemiology studies on the relationship between diabetes and infectious disease in SSA. In a region with a high burden of infectious disease, this has serious consequences for PLWD.

**Methods and findings:**

Using routinely collected longitudinal health data, we describe the epidemiology of diabetes in a large virtual cohort of PLWD who have a high burden of HIV and TB, from the Khayelitsha subdistrict in the Western Cape Province in South Africa. We described the relationship between previous TB, newly diagnosed TB disease and HIV infection on diabetes using HbA1c results as an outcome measure. The study population was predominately female (67%), 13% had a history of active TB disease and 18% were HIV positive. The HIV positive group had diabetes ascertained at a significantly younger age (46 years c.f. 53 years respectively, p<0.001) and in general had increased HbA1c values over time after their HIV diagnosis, when compared to the HIV-negative group. There was no evidence of TB disease influencing the trajectory of glycaemic control in the long term, but diabetes patients who developed active TB had higher mortality than those without TB (12.4% vs 6.7% p-value < 0.001). HIV and diabetes are both chronic diseases whose long-term management includes drug therapy, however, only 52.8% of the study population with an HIV-diabetes comorbidity had a record of diabetes treatment. In addition, the data suggest overall poor glycaemic control in the study population with only 24.5% of the participants having an HbA1c <7% at baseline despite 85% of the study population being on diabetes treatment.

**Conclusion:**

The epidemiologic findings in this exploratory study highlight the need for further research into diabetes outcomes in a high TB and HIV burden setting and demonstrate that routine health data are a valuable resource for understanding disease epidemiology in the general population.

## Introduction

Sub-Saharan Africa (SSA) is currently undergoing an epidemiologic shift and the health systems in the region are dealing with the dual burden of infectious diseases and an increasing prevalence of non-communicable diseases (NCDs) [1, 2]. NCDs are overtaking infectious disease as the leading cause of disability and mortality in the region [1, 3, 4]. This epidemiologic transition is already evident in South Africa where, although Tuberculosis (TB) was still the overall leading cause of natural deaths from 2015–2017, in the same time period, Diabetes Mellitus (DM) was the second leading cause of death [1]. The burden of DM is putting a strain on already struggling public health systems, and with an estimated 19 million people with diabetes in the region currently, projected to increase to 29 million by 2030, SSA is facing an impending diabetes epidemic [5].

More than 90% of diabetes in SSA is type 2 diabetes mellitus (T2DM) [6] which is thought to be largely fuelled by lifestyle changes brought about by a surge in rural-urban migration [7]. The diabetes epidemic in SSA including South Africa is further complicated by the ongoing HIV epidemic. South Africa is already implementing the UNAIDS 90-90-90 strategy which aims to get 90% of all those who test positive for HIV on anti-retroviral therapy (ART) [8]. This widespread use of ART has significantly increased the life expectancy of people living with HIV (PLWH), and the country is now supporting an aging HIV population that are developing comorbidities such as DM associated with aging which might also occur at earlier ages than in the general population [9, 10]. Studies have shown that in addition to demographic and lifestyle risk factors for DM the chronic use of ART, especially HIV protease inhibitors (PIs) and non-nucleoside reverse transcriptase inhibitors (NNRTIs), also contribute to the risk of developing DM [11–14].

While the widespread use of ART is reducing HIV/AIDS related morbidity and mortality—especially due to TB co-infection which is the leading cause of death in HIV positive people—it could potentially fuel the resurgence of diabetes-associated TB [8, 15–17]. The relationship between TB and DM is well established [18] and studies have shown that diabetes increases the risk of developing active TB, recurrent TB and severe TB disease and results in worse TB treatment outcomes [19–25]. The threat of a TB-DM dual epidemic in South Africa is a cause for concern given that the country is in the top eight highest TB burden countries, and in 2019 accounted for 3.6% of the global total of people who developed active TB [26], and the trilateral overlap with HIV may therefore have implications for TB control [27]. In addition, most of the DM in SSA including South African is undiagnosed until it presents with severe symptoms, so by the time most people get diagnosed they are already at risk of DM-related complications [28, 29].

It is widely accepted that people living with diabetes (PLWD) are at increased risk of infectious disease, yet there is a paucity of epidemiology studies on the relationship between diabetes and infectious disease in SSA. In a region with a heavy burden of infectious disease, this has serious consequences for PLWD in the region. Here, we describe the epidemiology of diabetes in a large virtual cohort of PLWD from the Western Cape Province in South Africa, who have a high burden of HIV and TB, using routinely collected longitudinal health data. We describe the relationship between previous and newly diagnosed TB disease and HIV infection and pre-existing diabetes using National Glycohemoglobin Standardization Program (NGSP) HbA1c as an outcome measure.

## Methods

### Ethics

Ethics approval was granted by the University of Cape Town (HREC REF: 509/2019) and data access was approved by Western Cape Government Health (WCGH), South Africa. All data were de-identified and data perturbation was employed by the Provincial Health Data Centre (PHDC, WCGH) prior to release, so that the data used were anonymised and cannot be reidentified. Data transfer was effected through secure platforms using AES256 encryption and password protection, and analysis was undertaken on a secured, firewall-protected server. Re-use of this dataset requires approval from the PHDC, and the authors can be contacted to advise on this process.

### Study population

**Fig 1:**
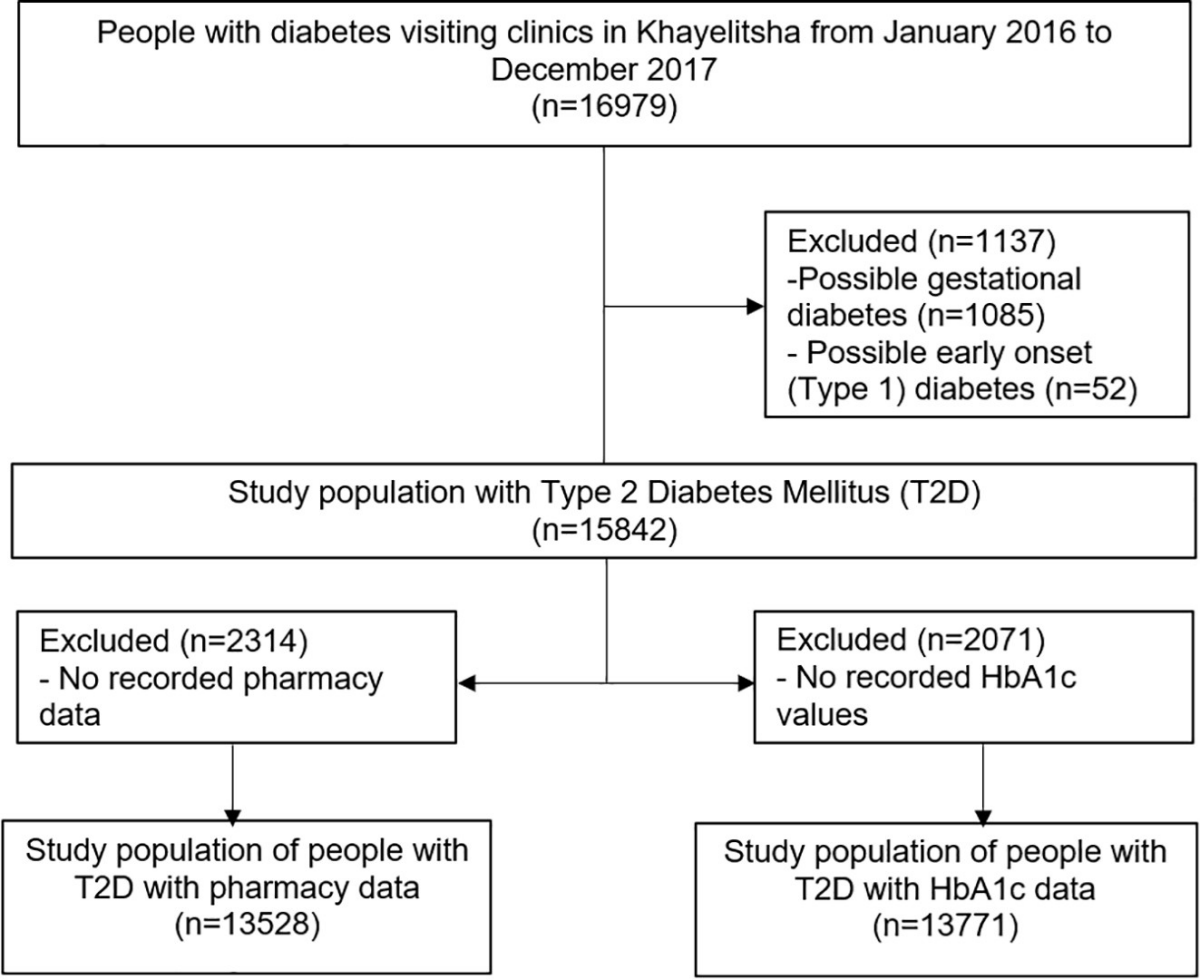
Flow chart showing the selection of the study population from the PHDC routine health data.

The study population was selected from the Western Cape Population as represented in the PHDC, a health information exchange containing routine health data for about 7 million healthcare clients, collated daily from multiple electronic health data sources in the Western Cape Province, South Africa [30]. Inclusion criteria were: (1) Having attended at least one Government Health Facility in the Khayelitsha sub-district in the Western Cape, South Africa, in the period 1 January 2016 to 31 December 2017, (2) aged 18 or older by December 2017 and (3) a diagnosis of diabetes inferred from PHDC records using listed disease evidences of at least one glycated haemoglobin (HbA1c) value greater than or equal to 6.5% [31], fasting glucose results, and/or dispensed diabetes drugs. The Khayelitsha subdistrict is a high-density urban area with large areas of informal housing and generally poor socioeconomic conditions. Exclusion criteria were: Diabetes ascertainment at less than 18 years of age, used as a proxy for early onset Type 1 Diabetes; and diabetes ascertainment occurring during pregnancy, used as a proxy for gestational diabetes (Fig 1). HbA1c is used as a diabetes outcome measure because it is the gold standard for diagnosing and monitoring diabetes control [31, 32], so people who did not have any recorded HbA1c values were also excluded from the analysis. HbA1c may underestimate glycaemia in PLWH but despite this remains highly specific for DM diagnosis [33]. When analysing medications, a subset was created which excluded those with no diabetes treatment records (Fig 1).

Retrospective PHDC data for 13 771 individuals with recorded HbA1c values were analysed together with population demographics (data as of 31 December 2017) using descriptive statistics. A diagnosis is inferred by the PHDC using laboratory and pharmacy data, and is not a clinical diagnosis, so is referred to as ‘ascertainment’ to make this distinction, and is described in [30]. Diabetes metrics include: Age at ascertainment, ‘Linkage to HbA1c testing’ was defined as having had a recorded HbA1c laboratory test result within one year of the last recorded diabetes-related health facility encounter, ‘Ever started diabetes treatment’ was defined as those with a recorded diabetes treatment start date and ‘linkage to diabetes treatment’ as those who had a record of diabetes drugs being prescribed within one year of their last recorded diabetes-related health facility encounter. ‘Baseline HbA1c’ was defined as the first recorded HbA1c value either at diabetes ascertainment or within the first year after diabetes ascertainment. TB metrics include: date of ascertainment of TB episodes for individuals from PHDC inferred episodes, ‘Ever had Tuberculosis’ was defined as having had a TB episode at any time in an individual’s recorded medical history and ‘TB-Diabetes comorbidity’ as having a recorded TB episode after Diabetes ascertainment. HIV metrics include the date of ascertainment of HIV from PHDC inferred episode data. HIV status was determined using date of HIV ascertainment and a record of initiation on HIV anti-retroviral therapy (ART).

Summary statistics were calculated for the study population. For continuous data, median and interquartile range were calculated and for grouped data, percentages were calculated. For median values, the Wilcoxon rank sum test was used to calculate significance of differences between groups; and significance of the differences in proportions between groups was tested using the Fisher’s exact test.

### TB and HIV comorbidities in the study population

New cases of Diabetes, TB and HIV were calculated for each year from 1^st^ January 2011 to 31^st^ December 2017. New Diabetes cases per year were counted as those with date of diabetes first ascertainment in that year, and this time range reflects a period during which most of the electronic data sources of the PHDC were in common use with relatively complete mortality data. New TB cases in each year were inferred from the PHDC episodes data as those where TB episode date was after date of diabetes first ascertainment. Likewise, new HIV cases were those where the date of HIV ascertainment was after diabetes ascertainment. The incidence of both TB and HIV per year in the study population over that 6-year period was calculated from these numbers.

Summary statistics describe the study population with a history of TB, comparing individuals with a TB episode before Diabetes was ascertained and those who developed active TB after their diabetes was ascertained (TB-Diabetes comorbidity). A person can have multiple cases of TB in their lifetime, and each time they are ascertained with TB it is recorded in the PHDC as a new TB episode with a start date and an end date. The ascertainment of TB episodes in relation to diabetes ascertainment was inferred using the episodes data to generate counts of the TB episodes of individuals before and after Diabetes ascertainment.

The HIV and TB status of the individual at the time of each HbA1c test was calculated and the ‘time to HbA1c ascertainment relative to TB ascertainment’ was inferred from the PHDC data by calculating the time difference in years between when each HbA1c test was done and when TB was ascertained in that individual. Negative time values were for HbA1c tests done before TB ascertainment, and positive time values were for HbA1c tests done after TB ascertainment.

The ‘time to HbA1c ascertainment relative to HIV ascertainment’ was inferred from the PHDC data by calculating the time difference in years between when each HbA1c test was done and when HIV status was ascertained in that individual. Negative time values were for HbA1c tests done before HIV ascertainment, and positive time values were for HbA1c tests done after someone is diagnosed as HIV positive.

### Diabetes treatment

Counts of the different diabetes drugs of individuals who had ever started diabetes treatment were done and stratified according to diabetes duration of the study participants. The Chi-squared test measured statistical significance in the difference in the proportions of people who were in the different groups.

## Results

### The study population

There were 16 969 individuals with an inferred diabetes episode, and of these, 15 842 were identified as most likely having Type 2 Diabetes Mellitus (T2DM) according to the described inclusion/exclusion criteria. Of the individuals with T2DM, 13 771 had recorded HbA1c laboratory results and 13 528 had pharmacy records for diabetes medications in the PHDC routine data (Fig 1). Of the study population, 67% had an average of one HbA1c test annually for the years assessed, although timing/spacing of the tests was not consistent.

Summary statistics (Table 1) show the study population was 67% female, with a median age at diabetes ascertainment of 52 years (IQR: 44, 59) and a 58% (N = 8003) people had been ascertained with diabetes less than 5 years. Diabetes is a progressive disease and we saw the median HbA1c was higher if the period since diabetes ascertainment was longer, with those ascertained more than 10 years previously having significantly higher HbA1c (Table 1). Almost everyone (>99%) who had had diabetes for more than 5 years was on diabetes treatments compared to only 75.1% (p<0.001) in those were ascertained less than 5 years earlier (Table 1). In addition, 18% were ascertained as HIV-positive, and there was no significant difference in the proportion of HIV-positive individuals when considering how long they have had diabetes. There was, however, a significant difference in the proportions of people who had a history of TB, where those who have had diabetes for less than 5 years had the lowest proportion (11.4%) and those who have had diabetes for 10 years or more having the highest proportion of people (17.9%). The same trend was observed for those who had a TB-Diabetes comorbidity, where 91.8% of those with a history of TB who have had diabetes 10 years or more had an active TB episode after being ascertained with diabetes (Table 1).

**Table 1:** Characteristics of the whole study population, and stratified by duration of diabetes in years.

	All *N = 13771*	0–5 Years *N = 8003*	5–10 Years *N = 5219*	≥ 10 Years *N = 549*	p-value
Sex (Female)	9246 (67.2%)	5225 (65.4%)	3635 (69.7%)	386 (70.4%)	<0.001
Age at diabetes ascertainment (Years)	52.0 [44.0;59.0]	52.0 [44.0;61.0]	51.0 [44.0;58.0]	50.0 [42.0;57.0]	<0.001
Baseline HbA1c (%) ^a^	8.5 [7.0;11.1]	7.9 [6.8;10.5]	9.5 [7.6;11.8]	9.9 [8.3;11.5]	<0.001
Last HbA1c (%)	9.5 [7.2;12.7]	8.6 [6.8;12.4]	10.3 [8.0;13.1]	10.8 [8.6;13.2]	<0.001
Patient outcome (Deceased)	631 (4.6%)	377 (4.7%)	237 (4.5%)	17 (3.1%)	0.213
Diabetes duration since ascertainment (Years)	4.1 [1.2;6.5]	1.6 [0.1;3.3]	6.6 [5.9;7.8]	10.7 [10.3;11.2]	0.000
Ever started diabetes treatment	11745 (85.3%)	6012 (75.1%)	5186 (99.4%)	547 (99.6%)	0.000
Ever had Tuberculosis	1839 (13.4%)	910 (11.4%)	831 (15.9%)	98 (17.9%)	<0.001
TB-Diabetes comorbidity ^b^	1008 (55.9%)	372 (41.9%)	547 (67.0%)	89 (91.8%)	<0.001
HIV Positive	2508 (18.2%)	1478 (18.5%)	932 (17.9%)	98 (17.9%)	0.657

a. Baseline HbA1c was defined as the first recorded HbA1c value either at diabetes ascertainment or within the first year after diabetes ascertainment

b. Proportions calculated from those who had ever had Tuberculosis

### TB and HIV in people living with diabetes

Comparing HIV-positive and HIV-negative groups showed people living with HIV (PLWH) had diabetes ascertained at a significantly younger median age than the HIV-negative population (46 years c.f. 53 years respectively, p<0.001) (Table 2). In addition, PLWH had a significantly higher most recent HbA1c than the HIV-negative population (12.1% c.f. 9.1%, p<0.001). In line with other findings, the percentage of people who have ever had TB was significantly higher amongst PLWH (32% vs 9%), but the proportion of HIV-negative individuals who developed active TB after diabetes ascertainment was significantly higher (62% c.f. 48%, p<0.001) than for PLWH. There was a significantly higher proportion of people with a history of TB in those who were ascertained with HIV before diabetes (35.9% c.f. 26.5%, p<0.001) compared to those who were ascertained HIV after diabetes.; and there was a significantly higher proportion of people with a TB-Diabetes comorbidity (88.5% c.f. 30.1%, p<0.001) in those who were ascertained with HIV after diabetes compared to those who were ascertained HIV before diabetes (S1 Table in S1 File). In addition, there was a significant difference in the percentage of people who were deceased when comparing those ascertained with HIV before or after diabetes ascertainment (4.5% c.f. 8.1%, p<0.001). This is unlikely to be only an effect of age, as the median ages at diabetes ascertainment in these two groups are 45.0 (IQR: 39.0, 52.0) c.f. 47.0 (IQR: 39.0, 53.0) years.

**Table 2:** Characteristics of the whole study population, and stratified by the HIV status of the participants.

	ALL *N = 13771*	HIV Negative *N = 11263* (82%)	HIV Positive *N = 2508* (18%)	p-value
Sex (Female)	9246 (67.2%)	7520 (66.9%)	1726 (68.9%)	0.054
Age at diabetes ascertainment (Years)	52.0 [44.0;59.0]	53.0 [46.0;61.0]	46.0 [39.0;52.0]	<0.001
Age categories				<0.001
18–39	2110 (15.3%)	1445 (12.8%)	665 (26.5%)	
40–49	3718 (27.0%)	2742 (24.3%)	976 (38.9%)	
50–59	4576 (33.2%)	3906 (34.7%)	670 (26.7%)	
60–69	2357 (17.1%)	2191 (19.5%)	166 (6.6%)	
70–79	805 (5.8%)	779 (6.9%)	26 (1.0%)	
> = 80	205 (1.5%)	200 (1.8%)	5 (0.2%)	
Baseline HbA1c (%)	8.5 [7.0;11.1]	8.6 [7.0;11.1]	8.4 [6.9;10.9]	0.008
Baseline HbA1c < 7%	2820 (24.5%)	2268 (24.0%)	552 (26.4%)	0.023
Last HbA1c (%)	9.5 [7.2;12.7]	9.1 [7.1;12.0]	12.1 [7.9;15.0]	<0.001
Last HbA1c < 7%	2928 (21.3%)	2509 (22.3%)	419 (16.7%)	<0.001
Patient outcome (Deceased)	631 (4.6%)	488 (4.3%)	143 (5.7%)	0.004
Ever started diabetes treatment	11745 (85.3%)	9631 (85.5%)	2114 (84.3%)	0.126
Linkage to diabetes ^a^ treatment	10707 (91.2%)	8913 (92.5%)	1794 (84.9%)	<0.001
Linkage to HbA1c testing ^b^	9264 (67.3%)	7580 (67.3%)	1684 (67.2%)	0.909
Ever had Tuberculosis	1839 (13.4%)	1039 (9.2%)	800 (31.9%)	<0.001
Tuberculosis-Diabetes comorbidity	1008 (55.9%)	627 (62.0%)	381 (48.2%)	<0.001

a. Proportions of patients having a record of diabetes drugs being prescribed within one year of their last recorded diabetes-related hospital encounter (calculated from those who had ever started diabetes treatment)

b. Proportion of patients having a recorded HbA1c laboratory test result within one year of the last recorded diabetes related hospital encounter

The TB population (S2 Table in S1 File) was 57% female with a median age at diabetes ascertainment of 49 years, and everyone in this cohort diagnosed with TB was linked to TB treatment. In addition, people with a history of TB had worse outcomes as we saw significantly more deceased people in this group when compared to those without a history of active TB disease (10% c.f. 3.8%, p<0.001). There was no significant difference in the gender distribution or age at diabetes ascertainment between those ascertained with TB before or after diabetes ascertainment (Table 3). The median baseline HbA1c of 10.1% (IQR: 7.6, 12.3) was significantly higher (p-value < 0.001) in those diagnosed with TB after diabetes when compared to those diagnosed with TB before diabetes at 8.2% (IQR: 6.8, 11.0). The results also suggest that developing active TB after a diabetes diagnosis may result in worse outcomes, as significantly more people in this group died (12%) when compared to those who had TB before being diagnosed with diabetes (7%). This is unlikely to be only an effect of age, as the median ages at diabetes ascertainment in these two groups are 49.0 (IQR: 41.2, 57.0) c.f. 48.0 (IQR: 41.0, 56.0) years.

**Table 3:** Characteristics of the study population with a history of Tuberculosis (TB) and stratified by the onset of the TB episode in relation to diabetes ascertainment.

	ALL N = 1802 (98%) ^a^	TB episode before diabetes ascertainment N = 794 (44%)	TB episode after diabetes ascertainment N = 1008 (56%)	p-value
Sex (Female)	1023 (56.8%)	442 (55.7%)	581 (57.7%)	0.415
Age at diabetes ascertainment (Years)	49.0 [41.0;56.0]	49.0 [41.2;57.0]	48.0 [41.0;56.0]	0.13
Baseline HbA1c (%)	9.2 [7.1;11.8]	8.2 [6.8;11.0]	10.1 [7.6;12.4]	<0.001
Baseline Hba1c < 7%	327 (22.3%)	189 (28.9%)	138 (17.0%)	<0.001
Last HbA1c (%)	11.0 [7.6;14.1]	10.8 [7.2;14.4]	11.1 [8.0;13.9]	0.130
Last HbA1c < 7%	328 (18.2%)	173 (21.8%)	155 (15.4%)	<0.001
HIV Positive	791 (43.9%)	410 (51.6%)	381 (37.8%)	<0.001
Diabetes duration since ascertainment (Years)	5.1 [1.8;7.1]	3.3 [0.5;6.1]	6.1 [3.5;7.9]	<0.001
Patient outcome (Deceased)	178 (9.9%)	53 (6.7%)	125 (12.4%)	<0.001
Ever started TB treatment	1802 (100.0%)	794 (100.0%)	1008 (100.0%)	.
Ever started diabetes treatment	1579 (87.6%)	640 (80.6%)	939 (93.2%)	<0.001
Linkage to diabetes treatment^b^	1350 (85.5%)	536 (83.8%)	814 (86.7%)	0.120
Linkage to HbA1c testing ^c^	1209 (67.1%)	532 (67.0%)	677 (67.2%)	0.983

a. 35 (2%) individuals who had ever had TB did not have enough data to classify when they had a TB episode relative to diabetes ascertainment

b. Proportions of patients having a record of diabetes drugs being prescribed within one year of their last recorded diabetes-related hospital encounter (calculated from those who had ever started diabetes treatment)

c. Proportion of patients having a recorded HbA1c laboratory test result within one year of the last recorded diabetes related hospital encounter

### Annual incidence of TB and HIV

**Fig 2:**
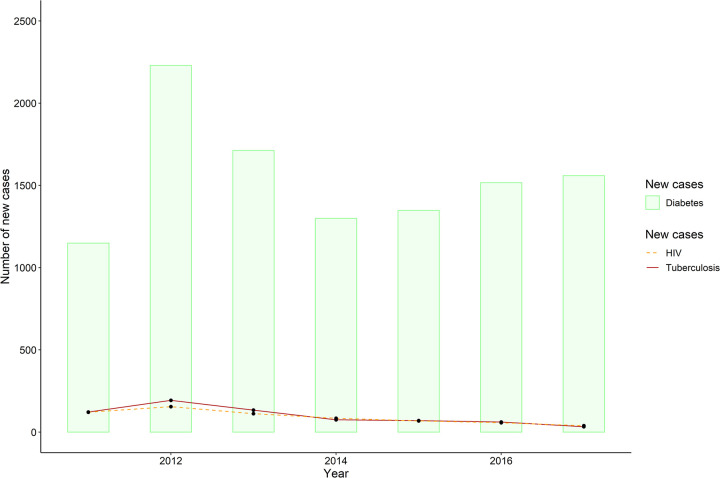
Bar graph showing new diabetes cases (bars) from January 2011 to December 2017 overlaid with line plots of new Tuberculosis (solid line) and HIV (dashed line) cases in these diabetes patients in the same time period.

New cases of Diabetes, TB and HIV were calculated in each year from 1^st^ January 2011 to 31^st^ December 2017. There was a steady increase of newly ascertained diabetes cases over the six-year period excluding 2012 and 2013 (Fig 2). The data also show there were almost equal numbers of new TB and HIV cases in the study population, and these numbers steadily decreased over the six-year period except for 2012 in which there was a spike for both. The TB and HIV incidence in this diabetes population were calculated at 1.06% per year and 0.98% per year respectively, calculated over the six-year period.

### Multiple episodes of TB

**Fig 3:**
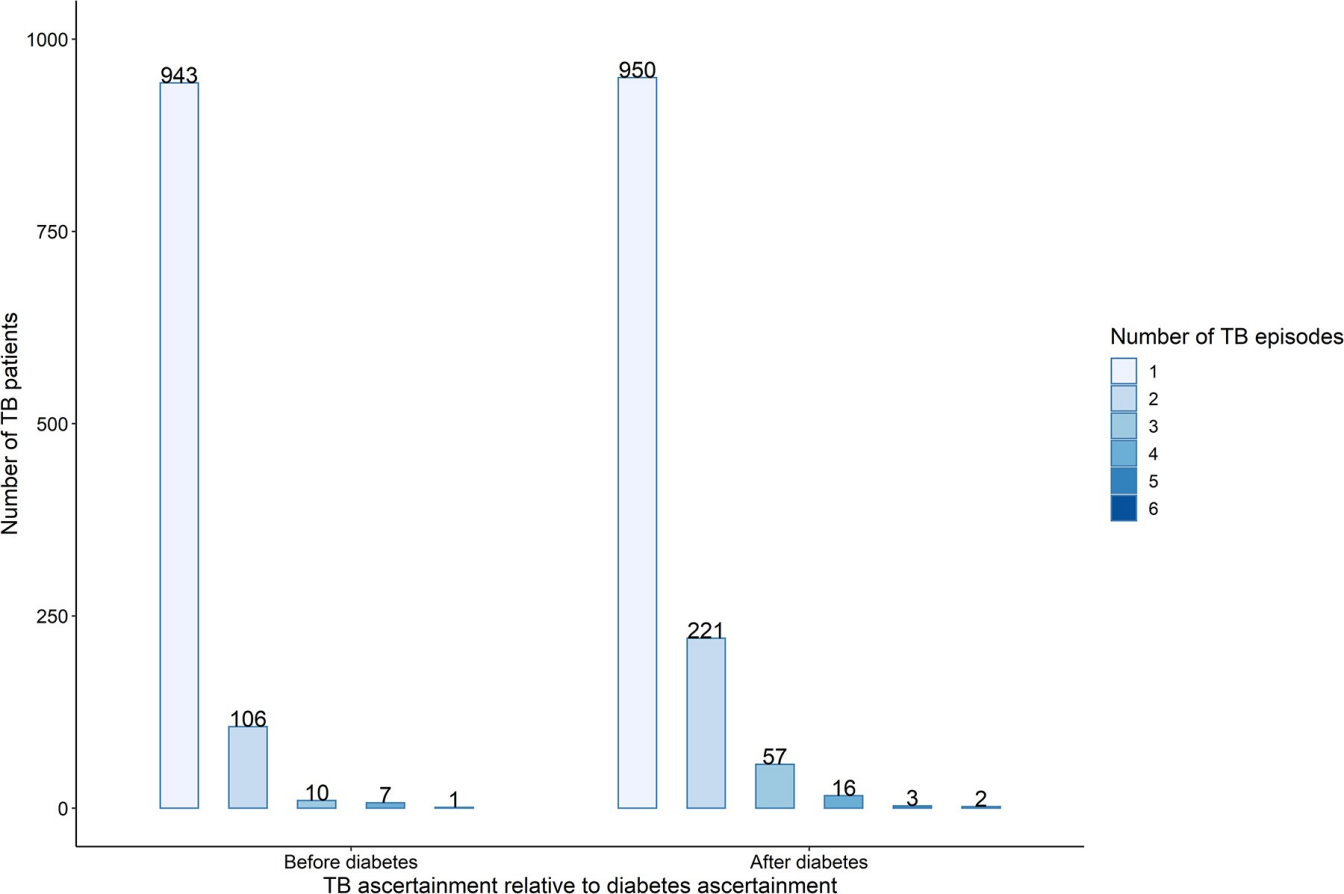
Distribution of repeat Tuberculosis (TB) episodes in the study population before and after diabetes ascertainment.

A person can have multiple cases of TB in their lifetime. Each time they are ascertained with TB it is recorded in the PHDC as a TB episode with start and end dates, and the ascertainment of TB episodes in relation to diabetes ascertainment was inferred using these data. There was a statistically significant difference (p-value < 0.001) in the distribution of TB episodes ascertained before and after diabetes ascertainment (Fig 3). The data show that after their first TB episode, significantly more people were getting subsequent TB episodes after diabetes ascertainment.

### HbA1c before and after TB ascertainment

**Fig 4:**
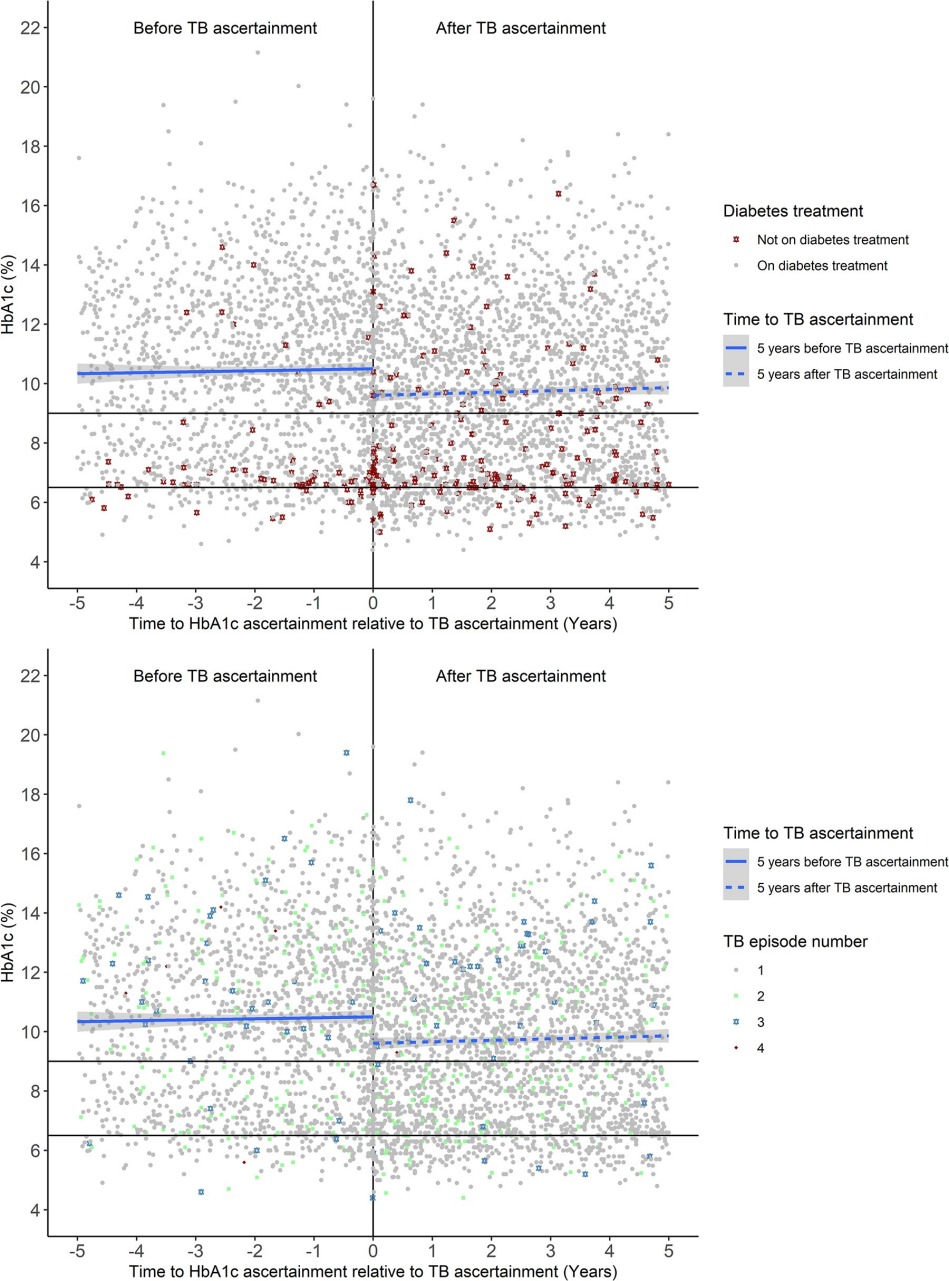
Effect of Tuberculosis ascertainment on HbA1c (%) over a 5-year period. A. HbA1c plotted by diabetes treatment i.e. on diabetes treatment (grey circle) or not on diabetes treatment (dark-red star). B. HbA1c plotted by TB episode i.e. 1 episode (grey circle), 2 episodes (pale-green square), 3 episodes (steel-blue star) or 4 episodes (dark-red diamond).

The overall mean population HbA1c measured during both the 5 years before and 5 years after TB ascertainment is greater than 9%, and is higher at the longer times since TB diagnosis, despite the majority of these patients receiving diabetes treatment (Fig 4A). Most of the HbA1c values of patients not on diabetes treatment are concentrated around an HbA1c of 6.5% which is the cut off HbA1c value for diagnosing diabetes, so it is reasonable to assume that these individuals are not yet receiving dispensed diabetes medications. Immediately after TB ascertainment, however, mean HbA1c is lower and there are more HbA1c values below 6.5% when compared to before TB ascertainment. In addition, after TB ascertainment, there are more recorded HbA1c values of patients not on diabetes treatment, and while most of these HbA1c values are concentrated around 6.5%, there are patients with HbA1c greater than 9% who are not on diabetes treatment. Both before and after TB ascertainment there is no distinct pattern for HbA1c values of patients who have had one or two TB episodes (Fig 4B), but for patients who have had three or four TB episodes the HbA1c values are mostly greater than 9%, and this is true for both before and after TB ascertainment. HbA1c values of participants who were deceased at study end were distributed randomly across the different HbA1c ranges both before and after TB ascertainment (S1 Fig in S1 File).

### HbA1c values with respect to HIV ascertainment

**Fig 5:**
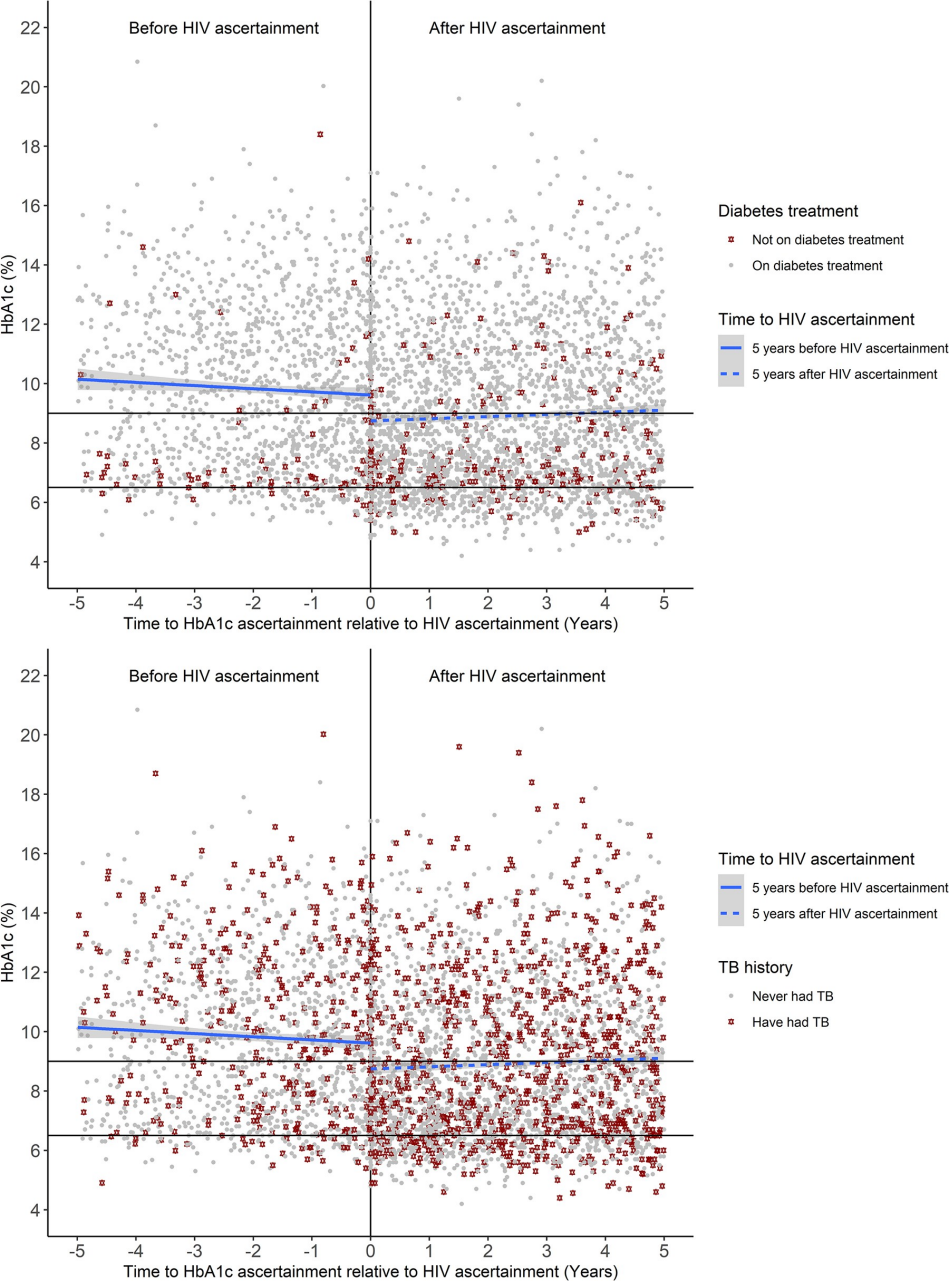
Effect of HIV ascertainment on HbA1c (%) over a 5-year period. A. HbA1c plotted by diabetes treatment i.e. on diabetes treatment (grey circle) or not on diabetes treatment (dark-red star). B. HbA1c plotted by TB history i.e. never had TB (grey circle) or have had TB (dark-red star).

The overall mean HbA1c measured during the 5 years before HIV ascertainment is greater than 9% but is generally lower at later time points and generally lowest (less than 9%) immediately after HIV ascertainment (Fig 5A). After HIV ascertainment, however, the overall mean HbA1c is generally a bit higher at later time points averaging just above 9%. Before HIV ascertainment the HbA1c values of those who are not on diabetes treatment are concentrated around the 6.5% diabetes diagnosis threshold, however after HIV ascertainment the HbA1c values of those not on diabetes treatment are distributed randomly across the different HbA1c values (Fig 5A). After HIV ascertainment, there were more recorded HbA1c values in individuals who have had TB, and the HbA1c values of those patients were distributed randomly across the different HbA1c ranges both before and after HIV ascertainment (Fig 5B). Similarly, HbA1c values of participants who were deceased at the study end were distributed randomly across the different HbA1c ranges both before and after HIV ascertainment (S2 Fig in S1 File).

### Diabetes treatment

The study population was dispensed the three main diabetes drug classes available in the National Formulary for the Public Sector: Metformin (MTF), Sulphonylurea (SU) and insulin (Table 4). In line with current treatment practices, most of the population were on oral drugs and the most widely prescribed drug was metformin for 95% of the population, with 41% of treatment patients on insulin. In addition, the use of Insulin increased significantly with increasing duration of diabetes with 79.6% of the people who have had diabetes for more than 10 years on insulin (Table 4). This result is in line with the high median HbA1c of patients, but even though 85% of the study population was on treatment, the HbA1c was generally high and also seemed to be higher at later timepoints after diagnosis—suggesting that diabetes is failing to be controlled the longer patients have had the condition (Table 1).

**Table 4:** Pharmacy counts with last recorded HbA1c values for the whole population and stratified by duration of diabetes in years since ascertainment.

	ALL *N = 13528*	0–5 Years *N = 7748*	5–10 Years *N = 5232*	≥ 10 years *N = 548*	p-value
Metformin	12702 (95.2%)	7137 (94.4%)	5051 (96.6%)	514 (93.8%)	<0.001
Sulphonylurea	8309 (62.3%)	3846 (50.9%)	4046 (77.4%)	417 (76.1%)	<0.001
Insulin	5513 (41.3%)	2012 (26.6%)	3065 (58.6%)	436 (79.6%)	0.000
Metformin & Sulphonylurea	8684 (64.2%)	4093 (52.8%)	4150 (79.3%)	441 (80.5%)	<0.001
Metformin & Insulin	5078 (37.5%)	1745 (22.5%)	2927 (55.9%)	406 (74.1%)	0.000
Metformin, Insulin & Sulphonylurea	3778 (27.9%)	1039 (13.4%)	2416 (46.2%)	323 (58.9%)	0.000

Many PLWD also had TB and HIV comorbidities, and while all the TB and HIV patients in this study were recorded as having started treatment for each disease respectively, not all diabetes patients were on treatment for diabetes. For the PLWD with TB and HIV comorbidities, only 59.5% (1088) of those with a TB-DM comorbidity were recorded as being on treatment for both TB and diabetes simultaneously, while only 52.5% (1323) of those with an HIV-DM comorbidity were recorded as being on both HIV and diabetes treatment simultaneously. Only 40.6% (743) of patients with a triple TB-HIV-DM comorbidity were recorded as being on treatment for all three conditions simultaneously (S3 Table in S1 File).

## Discussion

The study population was drawn from individuals visiting health care facilities with over-representation of women, in line with other reports showing men are less likely to seek health care compared to women, and there is a general bias due to physically healthy women linking to health care through contraceptive and maternal health programmes whereas health men seldom visit health facilities [34]. The distribution of people in the different age categories was similar for both men and women and the proportion of diabetes cases was highest at 33% in both men and women in the 50–59 age group (S4 Table in S1 File). A significantly higher proportion of HIV positive people had diabetes ascertained at less than 50 years of age (65.4% vs 37.1%; p-value < 0.001) when compared to those who were HIV-negative at diabetes ascertainment (Table 2). Whilst there may be a causal relationship between HIV and diabetes, it is also possible that HIV positive people may have earlier ascertainment of diabetes because they are accessing care frequently and therefore getting screened and diagnosed earlier rather than diagnosis only happening once they develop severe symptoms. Median baseline HbA1c was similar for HIV positive 8.4% (IQR: 6.9, 10.9) and HIV negative groups 8.6% (IQR: 7.0, 11.1), suggesting that PLWH may be presenting with similar diabetes severity to HIV-negative patients at diagnosis. T2DM is a disease that is associated with ageing, but when comparing the HIV-positive and HIV-negative groups we saw a significantly higher proportion of people PLWH who were between 18–39 years (26.5% c.f. 12.8% p-value < 0.001) being ascertained with T2DM (Table 2). This could be due to the interaction with HIV and Diabetes which increases the risk of diabetes and pre-diabetes in PLWH and especially those on highly active ART (HAART). There is also evidence that HIV significantly increases the risk of developing T2DM and that using highly active anti-retroviral therapy (HAART) induces hyperglycaemia [11–13], which is supported by our observations that, in a population with pre-existing diabetes, HIV co-infection appears in tandem with apparent glycaemic decline. We observed a median value of 8.4% (IQR: 6.9, 10.9) at baseline compared to 12.1% (IQR: 7.9, 15.0) at the last recorded HbA1c in this population, while in the HIV negative population there was only a slightly higher median of 9.1% (IQR: 7.1, 12.0) at the last recorded HbA1c compared to 8.6% (IQR: 7.0; 11.1) at baseline (Table 2). As all the HIV positive people in this study are on ART, the medications and the natural course of HIV infection might be contributing to the observed chronic hyperglycaemia. Other possibilities explanations include that HIV and diabetes care may not be well integrated in primary care clinics yet, and PLWH and DM may need to attend multiple clinics on multiple days leading to poor attendance.

The strong association between TB and HIV is well established and is reflected here with a TB burden in the HIV-positive population that is almost four times that in the HIV-negative population. Given the relationship between TB and HIV, a higher TB-Diabetes comorbidity in the HIV-positive group was expected, but we observed the opposite with significantly more HIV-negative people (62% c.f. 48.2%; p-value < 0.001) having a higher TB-Diabetes comorbidity (Table 1). This observation supports studies done in Nigeria [35] and Tanzania [36] which showed that HIV negative people living with diabetes had an increased risk of developing pulmonary TB than HIV-positive people living with diabetes. It is estimated that up to 80% of the population in South Africa is infected with *Mycobacterium tuberculosis* however, not everyone who is infected progresses to TB disease [37]. Studies have shown that in people with diabetes, the increased risk of TB disease is not necessarily from newly acquired infections, but rather by progression from latent to active TB [38], however the biological mechanisms have not yet been elucidated. It is possible that in our study, the significantly higher proportions of HIV-negative people with TB could be driven by progression from latent to active TB disease caused by diabetes especially given that this population group is not put on TB preventive therapy, while it is part of clinical care in PLWH in South Africa [37]. As the prevalence of diabetes continues to increase, it threatens to derail TB epidemic control efforts and there have been recent calls to assess the use of TB preventive therapy in people with diabetes [39, 40].

The relationship between T2DM and TB has been widely studied, but few studies have focused on the impact of active TB disease comorbidity on pre-existing diabetes. In this study we looked at the association between active TB disease and diabetes prognosis using HbA1c as an outcome. The target HbA1c for patients in care is 7% and as HbA1c levels increase so does the risk of diabetes complications [41]. Results from our study show that in people with pre-existing diabetes, overall mean HbA1c is highest in the year before TB ascertainment and lowest in the year after (Fig 4). A possible explanation for this observation in our study population could be that the participants were linked to diabetes care following TB diagnosis resulting in an improvement in their diabetes control. It is also possible that having a TB diagnosis and subsequent in these individuals might result in better control of diabetes and improved HbA1c levels once they are not TB-positive. Because our data are routine health data and do not include any clinician notes, however, we cannot conclude this from these data alone. Even though the HbA1c was generally lower after TB ascertainment, it was greater that > 9% overall which is still classified as uncontrolled diabetes. Our results are not comparable to many other studies [42–45] because most of these studies were cross sectional or had a short follow up time and did not report HbA1c before TB ascertainment. In addition, the studies investigated the impact of TB on the diagnosis of new diabetes and not on pre-existing diabetes. Overall, we observed that having TB disease did not seem to influence the trajectory of glycaemic control in the long term, but PLWD who developed active TB had worse outcomes, as we saw significantly more deaths (12.4% vs 6.7% p-value < 0.001) in this group (Table 2). Previous studies have shown that TB patients diagnosed with diabetes have worse TB outcomes [23, 46] and the same seems to hold true for TB patients with pre-existing diabetes. Since survival was the only patient outcome measure used in this study beyond HbA1c values, we could not determine the impact of the observed chronic hyperglycaemia on risk of developing diabetes related vascular complications which were observed in other studies [47].

HIV and diabetes are both chronic progressive illnesses which put a huge burden on the health care system [8], it is therefore important to understand how these two diseases affect each other in the South African context. While several studies have investigated the impact of HIV on glucose metabolism and the risk on developing pre-diabetes and diabetes [11–13], there is a paucity of studies investigating how HIV impacts the prognosis of pre-existing diabetes. In this study we aimed to investigate HbA1c levels in relation to new HIV infection in the context of pre-existing diabetes. Prior studies also show that HbA1c readings underestimate glycemia in HIV-infected individuals [48–50] and the results in our study might reflect these findings because we see a drop in mean HbA1c in the year following HIV ascertainment which only increases slightly over time. In addition, we also saw an overall trend in which HbA1c was lower before HIV ascertainment and this could be a possible indicator of undiagnosed HIV (Fig 5). It is also possible that the level of hyperglycaemia in PLWD who HIV are positive could be underestimated, suggesting that the utility of HbA1c in monitoring glycaemic control in HIV endemic settings like South Africa warrants further investigation.

T2DM can be managed using a combination of lifestyle changes and drug therapy and HbA1c levels are used as a proxy measure of long term diabetes control [32]. An HbA1c < 7% is the target level for good glycaemic control [51], however studies have shown that worldwide, people living with diabetes are failing to reach this glycaemic target [47, 52–55]. This study had similar results with only 24.5% (n = 2820) of the study population showing good glycaemic control at baseline. While this is worrying, it reflects that more than two thirds of diabetes in SSA including in South Africa is undiagnosed [28] until patients present with symptoms of chronic hyperglycaemia. The aim of diabetes management is controlling hyperglycaemia to reduce the risk of progression to microvascular and macrovascular complications [51], but this study indicated that this population is failing to reach this target despite 85% being recorded as being on treatment. This is a worrying trend which is possibly due to a combination of diabetes disease progression with time, and a lack of compliance and adherence with the treatment and lifestyle changes [54, 56–58]. Further analysis is needed to establish adherence and compliance in the study population, as this cannot be determined from the retrospective data alone.

HIV and diabetes are both chronic diseases whose long-term management includes drug therapy, however, only 52.8% of the study population with an HIV-diabetes comorbidity were on diabetes treatment. It is possible that there are both patient, provider and systems issues causing delay in initiation of therapy. Some patients might also get their diabetes care in the private health sector at different times during their care, and private health data were not included in this study, but it is unlikely that they would access public health facilities for one illness but not the other. These data suggest that a coordinated response is needed to address the gaps and provide an holistic, integrated care for people living with diabetes, especially in the context of the high burden of infectious diseases in Africa. Such an integrated approach would include education of PLWD, availability of health professionals with required skills, and sociodemographic considerations [59]. It will be important to better understand why almost 50% of patients with HIV-diabetes comorbidity are not on diabetes treatment despite the high median HbA1c suggesting a need for treatment intervention, and prospective studies can explore factors that determine treatment timelines especially with associated HIV diagnosis.

### Potential limitations of the study

There is a two-tier health system in South Africa where some individuals receive private health care, some receive only government health care, and there are also many individuals who access both types of service and transition back and forth depending on their employment and health insurance status [60, 61]. We therefore expect that an exhaustive health record for each individual may not be available through the PHDC. Some patients did not have recorded HbA1c results and pharmacy records, and this may could be due to private health service utilisation, as well as the staggered roll-out of electronic health data platforms in the Province which means that data completeness may fluctuate according to the facility attended and year of service provision. Also, South Africa has a federated health service whereby provinces manage healthcare services [60, 61], and coupled with a highly migratory working population, it is possible that records are missing when individuals move to other provinces in South Africa for periods of time.

## Conclusion

To our knowledge this is the first study in South Africa to use longitudinal routine health data to study the relationship between active TB disease and HIV infection in the context of pre-existing diabetes using National Glycohemoglobin Standardization Program (NGSP) HbA1c as an outcome measure. In addition, we were able to establish temporal order of disease ascertainment. The study had a large sample size and long-term retrospective data, reducing selection bias arising from including people actively seeking care. In addition, these routine health data reflect a more accurate picture of diabetes in the general population than would actively managed clinical studies involving diabetes patients. The epidemiologic findings in this exploratory study demonstrate that routine health data are a valuable resource for understanding disease epidemiology and highlighted the need for further research into diabetes outcomes in a high TB and HIV burden setting.

## Supporting information

S1 File (PDF)
