# Supplementary material for: Diabetes in a TB and HIV-endemic South African population: Analysis of a virtual cohort using routine health data
Source: PLoS One. 2021 May 7;16(5):e0251303. doi: 10.1371/journal.pone.0251303 (PMC8104376; doi:10.1371/journal.pone.0251303)
Supplement: S1 File — (PDF) [file pone.0251303.s001.pdf]

Supplementary data:

Diabetes in a TB and HIV-endemic South African population: Analysis of a virtual cohort using routine health data.

S1 Table: Characteristics of the study population who are HIV positive and stratified by the ascertainment of the HIV in relation to diabetes ascertainment

	**All** *N=2423*[a]	**HIV before diabetes ascertainment** *N=1525 (63%)*	**HIV after diabetes ascertainment** *N=898 (37%)*	p-value
Sex (Female)	1676 (69.2%)	1033 (67.8%)	643 (71.6%)	0.058
Age at diabetes ascertainment (Years)	46.0 [39.0;52.0]	45.0 [39.0;52.0]	47.0 [39.0;53.0]	0.029
Baseline HbA1c (%)	8.4 [6.9;10.9]	8.0 [6.8;10.4]	9.4 [7.3;11.8]	<0.001
Last HbA1c (%)	12.2 [8.0;15.0]	12.5 [7.9;15.2]	11.6 [8.0;14.5]	0.005
Patient outcome (Deceased)	142 (5.9%)	69 (4.5%)	73 (8.1%)	<0.001
Diabetes duration (Years)	4.0 [1.2;6.5]	2.9 [0.6;5.6]	5.8 [3.1;7.8]	<0.001
Ever started diabetes treatment	2056 (84.9%)	1237 (81.1%)	819 (91.2%)	<0.001
Ever had Tuberculosis	786 (32.4%)	548 (35.9%)	238 (26.5%)	<0.001
TB-Diabetes comorbidity	372 (47.8%)	164 (30.1%)	208 (88.5%)	<0.001

a. 85 (3%) individuals did not have enough data to classify when they were ascertained HIV relative to diabetes ascertainment

S2 Table: Characteristics of the whole study population, and stratified by history of active Tuberculosis disease

	**ALL** *N=13771*	**Never had Tuberculosis** *N=11932*	**Ever had Tuberculosis** *N=1839*	**p-value**
Sex (Female)	9246 (67.2%)	8201 (68.8%)	1045 (56.9%)	<0.001
Age at diabetes ascertainment (Years)	52.0 [44.0;59.0]	52.0 [44.0;60.0]	49.0 [41.0;56.0]	<0.001
Baseline HbA1c (%)	8.5 [7.0;11.1]	8.5 [7.0;11.0]	9.2 [7.1;11.8]	<0.001
Last HbA1c (%)	9.5 [7.2;12.7]	9.3 [7.1;12.5]	10.9 [7.6;14.1]	<0.001
Patient outcome (Deceased)	631 (4.6%)	448 (3.8%)	183 (10.0%)	<0.001
TB-Diabetes comorbidity	1008 (55.9%)	0 (.%)	1008 (55.9%)	.
Ever started TB treatment	1831 (13.3%)	0 (0.0%)	1831 (99.6%)	0.000
Ever started diabetes treatment	11745 (85.3%)	10141 (85.0%)	1604 (87.2%)	0.013
Linkage to diabetes Treatment	10707 (91.2%)	9335 (92.1%)	1372 (85.5%)	<0.001
Linkage to HbA1c testing	9264 (67.3%)	8029 (67.3%)	1235 (67.2%)	0.896

S3 Table: Number of study participants recorded as being on the different combinations of Diabetes, TB, and HIV medications.

Diabetes treatment	Tuberculosis treatment	HIV treatment	Number of study participants
1	0	0	10374
1	0	1	1323
1	1	0	1088
1	1	1	743

S4 Table: Characteristics of the whole study population, and stratified by the sex of the participants

	**[ALL]** *N=13754* [a]	**Female** *N=9246* (67%)	**Male** *N=4508* (33%)	**p-value**
Age at diabetes ascertainment (Years)	52.0 [44.0;59.0]	52.0 [44.0;60.0]	51.0 [43.0;59.0]	<0.001
Age categories				<0.001
18-39	2108 (15.3%)	1348 (14.6%)	760 (16.9%)	
40-49	3711 (27.0%)	2476 (26.8%)	1235 (27.4%)	
50-59	4570 (33.2%)	3090 (33.4%)	1480 (32.8%)	
60-69	2356 (17.1%)	1597 (17.3%)	759 (16.8%)	
70-79	804 (5.8%)	569 (6.2%)	235 (5.2%)	
>=80	205 (1.5%)	166 (1.8%)	39 (0.9%)	
HIV Positive	2506 (18.2%)	1726 (18.7%)	780 (17.3%)	0.054
Ever had Tuberculosis	1838 (13.4%)	1045 (11.3%)	793 (17.6%)	<0.001
Tuberculosis-Diabetes comorbidity	1007 (55.9%)	581 (56.8%)	426 (54.8%)	0.415
Baseline HbA1c (%)	8.5 [7.0;11.1]	8.6 [7.0;11.1]	8.5 [6.9;11.2]	0.441
Baseline HbA1c < 7%	2816 (24.5%)	1867 (24.0%)	949 (25.5%)	0.089
Last HbA1c (%)	9.4 [7.2;12.7]	9.5 [7.2;12.7]	9.3 [7.1;12.7]	0.038
Last HbA1c < 7%	2927 (21.3%)	1886 (20.4%)	1041 (23.1%)	<0.001
Patient outcome (Deceased)	631 (4.6%)	394 (4.3%)	237 (5.3%)	0.010
Ever started TB treatment	1830 (13.3%)	1043 (11.3%)	787 (17.5%)	<0.001
Ever started diabetes treatment	11729 (85.3%)	7802 (84.4%)	3927 (87.1%)	<0.001
Linkage to diabetes treatment	10694 (91.2%)	7117 (91.2%)	3577 (91.1%)	0.838
Linkage to HbA1c testing	9250 (67.3%)	6157 (66.6%)	3093 (68.6%)	0.021

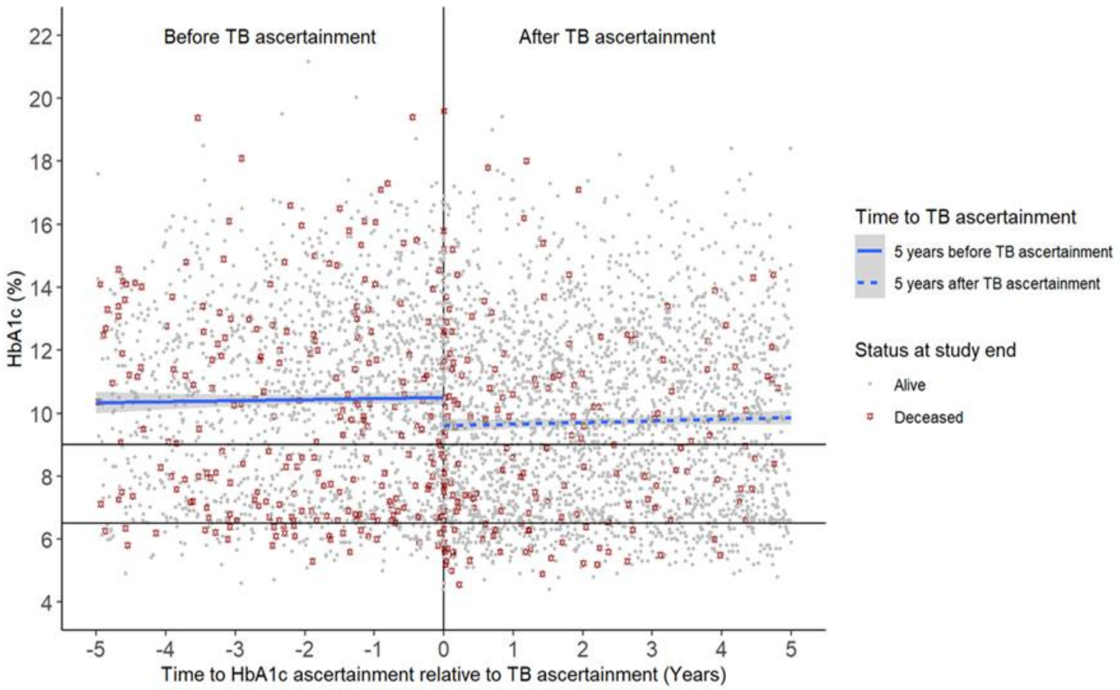

S1 Fig: Effect of Tuberculosis ascertainment on HbA1c (%) over a 5-year period**.** HbA1c plotted by participant outcome at the end of the study i.e. alive (grey circle) or deceased (dark-red star).

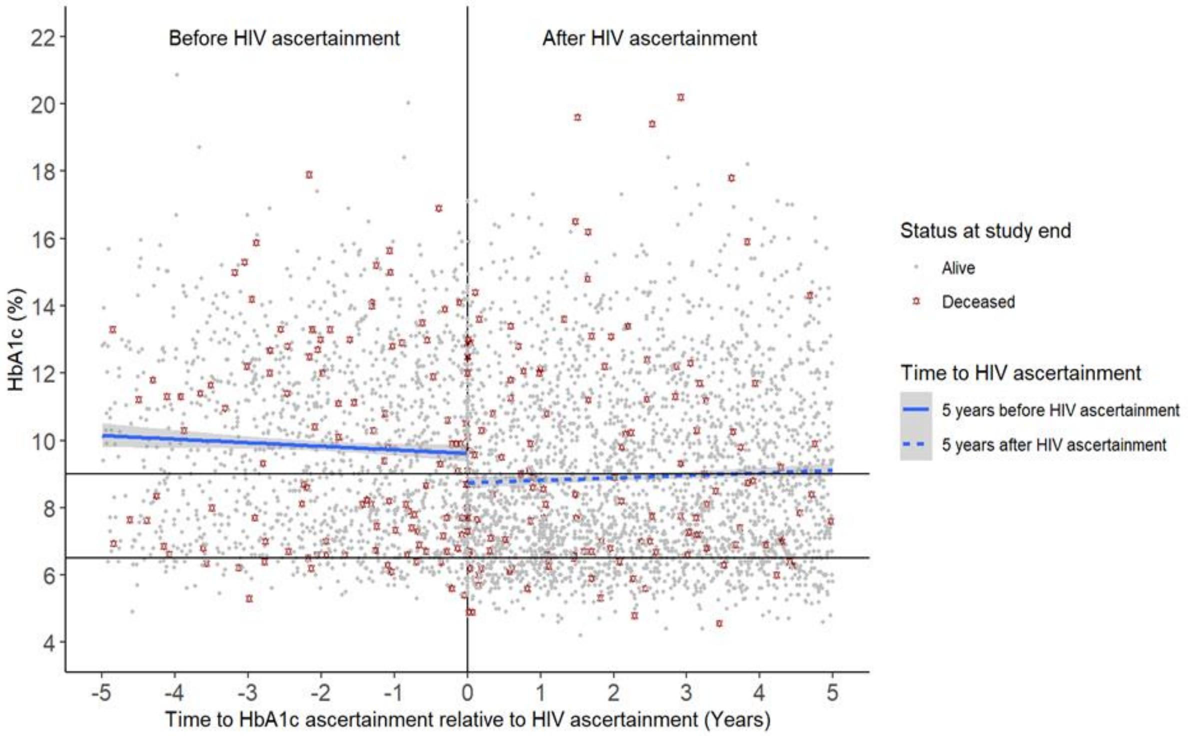

S2 Fig: Effect of HIV ascertainment on HbA1c (%) over a 5-year period**.** HbA1c plotted by participant outcome at the end of the study i.e. alive (grey circle) or deceased (dark-red star).
